# Measuring positive affect and well-being after spinal cord injury: Development and psychometric characteristics of the SCI-QOL Positive Affect and Well-being bank and short form

**DOI:** 10.1179/2045772315Y.0000000024

**Published:** 2015-05

**Authors:** Hilary Bertisch, Claire Z. Kalpakjian, Pamela A. Kisala, David S. Tulsky

**Affiliations:** 1 Rusk Rehabilitation, New York University Langone Medical Center, New York, NY, USA; 2 Department of Physical Medicine and Rehabilitation, University of Michigan Medical School, University of Michigan, Ann Arbor, MI, USA; 3 Department of Physical Therapy, University of Delaware, College of Health Sciences, Newark, DE, USA; 4 Kessler Foundation Research Center, West Orange, NJ, USA

**Keywords:** Affect, Spinal Cord Injuries, Patient Outcomes Assessment, Quality of Life, Psychometrics, Emotions

## Abstract

**Objective:**

To develop an item response theory (IRT)-calibrated spinal cord injury (SCI)-specific Positive Affect and Well-being (PAWB) item bank with flexible options for administration.

**Design:**

Qualitative feedback from patient and provider focus groups was used to expand on the Neurological Disorders and Quality of Life (Neuro-QOL) positive affect & well-being item bank for use in SCI. New items were created and revised based on expert review and patient feedback and were then field tested. Analyses included confirmatory factor analysis, graded response IRT modeling and evaluation of differential item functioning (DIF).

**Setting:**

We tested a 32-item pool at several rehabilitation centers across the United States, including the University of Michigan, Kessler Foundation, Rehabilitation Institute of Chicago, the University of Washington, Craig Hospital and the James J. Peters/Bronx Department of Veterans Affairs hospital.

**Participants:**

A total of 717 individuals with SCI answered the PAWB questions.

**Results:**

A unidimensional model was observed (Confirmatory Fit Index = 0.947; Root Mean Square Error of Approximation = 0.094) and measurement precision was good (reliability in theta of –2.9 to 1.2 is roughly equivalent to classical reliability of 0.95 or above). Twelve items were flagged for DIF, however, after examination of effect sizes, the DIF was determined to be negligible and would have little practical impact on score estimates. The final calibrated item bank resulted in 28 retained items

**Conclusions:**

This study indicates that the Spinal Cord Injury – Quality of Life PAWB bank represents a psychometrically robust measurement tool. Short form items are also suggested and a computer adaptive test is available.

## Introduction

Psychological outcomes research in spinal cord injury (SCI) has typically focused psychopathology.^1–8^ Recently, however, there has been a growing recognition of the value of examining positive characteristics and outcomes following SCI, reflecting an appreciation for the diversity of emotional responses to injury.^9,10^ In qualitative studies, individuals with SCI report themes related to positivity and growth subsequent to their trauma.^11^ Still, many questions remain about positive psychological factors that lead to improved outcomes after injury and how they may be enhanced in rehabilitation settings.

A catalyst of the recent interest in studying positive psychological variables in the context of SCI is the expansion of the field of ‘positive psychology,’ first introduced by Martin Seligman and Mihaly Csikszentmihalyi in 2000.^12^ In contrast to traditional models of psychological functioning that emphasize pathology, positive psychology highlights attributes such as positive affect, well-being, resilience, optimism, hope, and perseverance.^12,13^ Inclusion of these characteristics into conceptual models of psychological functioning after trauma provides a more complete picture that can guide treatment models and classification systems in the context of rehabilitation.^13^ Emerging evidence suggests that the study of positive affect, well-being, and resilience is particularly relevant to individuals who sustained SCI.^14–23^ Research suggests that trajectories for recovery may not be a linear process but rather involve more complex interactions between stage of recovery, demographics, and importantly, cognitive appraisals.^14–17^ Because factors such as positive affect, well-being, resilience, optimism, and hope have been shown to improve adaptation and outcomes in persons with SCI,^15,18–23^ their facilitation in the rehabilitation process is an important target of examination and intervention.

As the study of positive psychology variables continues to emerge within in SCI research, it has been increasingly necessary to distinguish between the distinct constructs within the field in order to improve the clarity of results within and across studies. Early work in SCI research focused on the important roles of adjustment and life-satisfaction following injury, and this body of research continues to develop.^24–35^ More recently, the study of resilience in SCI research has significantly expanded, but a single operational definition for this term that extends across studies is still being established.^19,21,22,36^ Other work has provided evidence for the benefits of attributes such as self-efficacy, self-esteem, spirituality, optimism, and hope in individuals with SCI, but this body of literature has been described as ‘broad, but fragmented’^9^ which may be related in part to a lack of consistent terminology leading to an overlap between determinants and outcomes, as well as inconsistency in use of measurement tools.^10^

The challenges of operationalizing positive affect and well-being in the context of SCI research are related, in part, to the limited availability of appropriate instruments for assessing these constructs and the inconsistent use of measurements across studies.^11,15^ The Connor-Davidson Resilience Scale is widely used in studying positive psychological outcomes, however this scale is primarily a measure of resilience, and places less emphasis on overall positive affect, well-being, and other related characteristics.^19,36–39^ Moreover, there can be subtle differences between the quality of resilience and attributes such as positive affect and well-being; such nuances may have differential impact on outcomes. A variety of instruments including the Personal Well-being Index,^17^ the Life Orientation Test-Revised, the Positive Affect and Negative Affect Schedule,^23^ the Intrinsic Spirituality Scale,^38^ the Life Satisfaction Questionnaires,^16^ and the Spinal Cord Lesion Emotional Well-being Scale,^40^ have been utilized in SCI studies to capture characteristics like positive affect and well-being, but many of these instruments have not been well-validated in SCI populations. Other studies have assessed positive characteristics utilizing simple interview questions, further contributing to the heterogeneity of measurement across studies.^15,21,41^ Just as clearer operational definitions are needed to inform measurement, validity in measurement is also necessary to increase precision in defining constructs such as positive affect and well-being within SCI research.

Addressing these current limitations in measurement of positive psychological outcomes in SCI, the purpose of this paper is to present findings from the development and psychometric calibration of the SCI-QOL Positive Affect & Well-being item bank and short forms

## Methods

This study was approved by all participating sites’ Institutional Review Boards. The first study activity was to develop and refine a positive affect and well-being item pool. Next, items were administered to a large sample of people with SCI using a computerized data collection platform and interview format, so that each question was read to the respondent by a trained interviewer and responses were directly entered into the database. Each of these steps is described in detail in Tulsky *et al*. and is also outlined briefly in the section below.

### Development of a positive affect & well-being item pool

To develop the positive affect and well-being item bank, we began by identifying candidate items from our initial pilot work, which included individual, semi-structured interviews and focus groups with patients with SCI and clinicians who specialize in SCI medicine (see Tulsky *et al*.^11^ for a full description). From the interview data, we developed a set of 51 preliminary items related to positive affect and well-being. Specific phrases or concepts were then drawn from the focus group transcripts and converted into 28 additional ‘new’ items. For example, a focus group participant with paraplegia stated, ‘*I never thought that…I could overcome this as much as I have in the past 6 and a half years,*’ and from that quote we drafted the item, ‘I was proud of everything that I have overcome.’ Twenty-three more items were drawn from the Neuro-QOL measurement system; all wording was retained verbatim. Many of the Neuro-QOL items were redundant with the new items created from interviews and focus groups. In these cases, if the overlap was deemed sufficient, the new items were dropped in favor of the Neuro-QOL items to maintain consistency.

The initial 70 items then underwent Expert Item Review (EIR),^42^ a method whereby several project co-investigators reviewed each item for relevance and clarity and made suggestions for revisions and deletions. Based on EIR feedback, 48 items were retained in the preliminary positive affect and well-being item pool. Preliminary items then underwent an additional phase of item review and modification by members of the investigative team. Items were arranged on a hierarchy of ‘difficulty’, from items indicating the lowest degree of positive affect and well-being to the highest degree of positive affect and well-being. Team members removed redundant items where there was oversaturation in the middle range of the hierarchy, and, if necessary, suggested new items to fill gaps in content coverage. During this phase of review, an additional 16 items were removed.

With the exception of the 27 items originally from Neuro-QOL which already underwent cognitive debriefing, this refined set of positive affect and well-being items was then evaluated with individuals with SCI during structured cognitive debriefing interviews.^43^ These required participants to answer each item, then describe the process they used to come up with their answer and relate whether they perceived anything to be confusing, unclear, or derogatory, or whether they thought any items could be better phrased. One item was modified and no items were deleted based on cognitive interviewing. After this phase, the final 5 new (i.e. not originally from Neuro-QOL) items were reviewed for translatability (for method, please see Eremenco *et al*.)^44^ and reading level (using the Lexile framework).^45^ Slight modifications were made to 2 items after the translatability and cultural review. The item ‘I was optimistic about things to come’ was changed to ‘I was optimistic about the future,’ since ‘things to come’ would be ambiguous if translated in this context, and the item ‘I was proud of how much I have overcome’ was changed to ‘I was proud of everything that I have overcome’ since this would be easier and more natural to say in Spanish. All items were written at the 5^th^ grade reading level.

### Calibration study participants and data collection procedures

As a part of a large-scale multisite item calibration study (sites included the Kessler Foundation, University of Michigan, Rehabilitation Institute of Chicago, University of Washington, Craig Hospital and the James J. Peters/Bronx Veterans Administration hospital), we administered the initial 32 positive affect and well-being items along with other item pools reflecting different Health Related Quality of Life (HRQL) subdomains to a sample of people with SCI.

The calibration sample included 717 participants with SCI. Inclusion criteria were 18 years of age and older, ability to read and understand English, and medically documented traumatic SCI. The sample was stratified by level (paraplegia versus tetraplegia), completeness of injury (complete vs. incomplete), and time since injury (<1 year, 1–3 years, and >3 years) to ensure that the final sample was a heterogeneous sample of individuals with SCI. Each participant's diagnosis was confirmed by medical records and each participant's neurologic level was documented by their most recent American Spinal Injury Association Impairment Scale (AIS) rating. All items were presented in a structured interview to participants in person or over the phone. The methodology for this study is presented in detail in Tulsky *et al*.^50^ and will not be repeated here.

#### Data analyses

Analysis involved confirmation of construct unidimensionality, use of a graded-response IRT model^46^ to calibrate item parameters, and examination of differential item functioning. We used confirmatory factor analyses to determine if our items conformed to a unidimensional model. Acceptable model fit indices were: CFI > 0.90, CFI > 0.95 = excellent^47^; RMSEA < 0.08, good, RMSEA < 0.06, excellent.^48,49^ Calibration was performed using iterative methods to reduce the item pool and obtain the best-fitting item parameters that would best allow estimation of a participant's standing on a trait of positive affect and well-being. With each successive analytic iteration, we identified poorly fitting items by examining item fit to the 2-Parameter Linear (PL) IRT model, DIF, local dependence between items (residual correlations >|0.20|), and significant loadings on the single factor (values >0.30). We then removed these items from the item pool and repeated the analytic steps. Once an acceptable solution was reached with CFA statistics that supported a unidimensional model, and all items showing misfit to the model or DIF were removed, the bank was finalized. Next, the SCI-specific IRT parameters were transformed to the Neuro-QOL metric (which was calibrated in a general population sample) using the Stocking and Lord procedure as described by Tulsky *et al*.^50^ These final transformed IRT parameters were utilized to develop a computerized adaptive test (CAT) version of the bank. The CAT was programmed on the Assessment Center website (www.assessmentcenter.net) and can be administered directly from there. The final (transformed) parameters were also used to select items for a static short form which can also be downloaded as a Portable Document Format (PDF) from the Assessment Center website. Tulsky *et al*.^50^ within this special issue described the detailed methodology and data analysis plan. PDF copies of the item bank and short form are also available from the corresponding author.

## Results

### Participant characteristics

Positive affect and well-being items and other item pools were administered to a calibration sample of 717 individuals with SCI. Demographic and injury characteristics are summarized in Table 1. Please see the Tulsky *et al*.^51^ introductory article within this special issue for additional details on the calibration sample, including education, income level and mechanism of injury.

**Table 1:** Calibration sample – participant characteristics

Variable	Emotional domain sample, *N* = 717 mean (SD), *N* (%)
Age (years)	43.0 (15.3)
Age at injury (years)	36.1 (16.8)
Sex	
Male	559 (78%)
Female	158 (22%)
Ethnicity	
Hispanic	82 (11%)
Non-Hispanic	631 (88%)
Race	
Caucasian	505 (70%)
African-American	125 (17%)
Asian	8 (1%)
American Indian/Alaska Native or Native Hawaiian/Pacific Islander	7 (1%)
More than one race	9 (1%)
Other	50 (7%)
Time Since Injury	7.1 (10.0)
<1 year post injury	196 (27%)
1–3 years post injury	186 (26%)
>3 years post injury	335 (47%)
Diagnosis	
Paraplegia complete	182 (25%)
Paraplegia incomplete	143 (20%)
Tetraplegia complete	157 (22%)
Tetraplegia incomplete	231 (33%)

### Preliminary analysis and item removal

Data analysis began with the full pool of 32 items Following the first iteration of preliminary analyses and CFA, 3 items were removed due to local item dependence (LID) and/or low item-total correlation. Additionally, one item that was also deleted from Neuro-QOL was removed due to poor wording/double-barreled language (NQPPF27 *‘I felt loved and wanted’*). The following results are based on the final 28-item set. Of the 28 items, 22 are final Neuro-QOL items, 1 item (NQPPF01) was originally in Neuro-QOL but deleted during calibration, and 5 items were newly written during the initial qualitative phase of the SCI-QOL project.

For the final 28 items, internal consistency was α = 0.970 and item/total correlations ranged from 0.61 to 0.82. All of the items but one (NQPPF23) had more than 20% of the sample selecting category 5 (Always). Three items had a category inversion with the average raw score for persons selecting category 2 (Rarely) lower than the average for person selecting category 1 (Never). However, these category inversions occurred when there were very few respondents endorsing the extreme categories (i.e. Never or Always). The disordinal mean scores were based on small n-counts and hence considered negligible and localized when the global indices (e.g. item-total correlation, IRT slope parameter, IRT fit) did not reveal any anomalies. No further items were removed at this stage. Descriptive statistics for each of the final items are provided in Table 2.

**Table 2:** Descriptive item statistics

Item ID	Item stem	Mean	SD	% at min	% at max
NQPPF01	I felt happy about the future	3.71	1.104	3.8	29.5
NQPPF02	I was able to enjoy life	3.90	0.990	1.8	32.2
NQPPF03	I felt a sense of purpose in my life	3.98	1.063	2.4	40.7
NQPPF04	I could laugh and see the humor in situations	4.14	0.895	1.0	41.1
NQPPF05	I was able to be at ease and feel relaxed	3.63	1.033	2.7	23.5
NQPPF06	I looked forward with enjoyment to upcoming events	3.97	1.002	1.4	37.4
NQPPF07	Many areas of my life were interesting to me	3.77	1.040	1.8	30.1
NQPPF08	I felt emotionally stable	4.01	0.961	2.0	35.8
NQPPF11	I felt confident	3.80	1.015	2.2	28.6
**NQPPF12**	**I felt hopeful**	4.01	0.965	2.2	36.6
NQPPF13	I had a good life	3.98	1.017	2.4	37.6
**NQPPF14**	**I had a sense of well-being**	3.85	1.007	2.1	30.8
NQPPF15	My life was satisfying	3.70	1.027	2.8	24.7
**NQPPF16**	**I had a sense of balance in my life**	3.61	1.053	3.1	24.0
**NQPPF17**	**My life had meaning**	3.99	1.083	2.9	42.8
NQPPF18	My life was peaceful	3.61	1.046	3.5	22.2
**NQPPF19**	**My life was worth living**	4.48	0.842	1.4	65.2
**NQPPF20**	**My life had purpose**	4.08	1.070	2.8	47.3
**NQPPF21**	**I was living life to the fullest**	3.45	1.218	7.0	25.5
**NQPPF22**	**I felt cheerful**	3.74	0.928	1.4	22.5
NQPPF23	In most ways my life was close to my ideal	2.89	1.211	15.9	10.9
NQPPF24	I had good control of my thoughts	4.05	0.919	1.7	36.0
NQPPF26	Even when things were going badly, I still had hope	4.16	0.929	1.4	44.9
PPF_29	I was optimistic about the future	3.78	1.080	3.2	31.4
**PPF_30**	**I thought positively about my future**	3.91	1.059	3.1	36.5
**PPF_32**	**I was thankful to be alive**	4.48	0.881	1.5	67.0
PPF_33	I was proud of everything that I have overcome	4.13	0.982	1.7	45.8
PPF_34	I had a positive attitude about living with my injury	3.87	1.040	3.5	32.0

*Context for all items was: ‘Lately’. Response set was: 1 = Never/2=Rarely/3 = Sometimes/4 = Often/5 = Always.

**Bold Font** indicates the items selected for the short form 10a.

With the exception of item NQPPF01, all items beginning with ‘NQPPF’ are final Neuro-QOL PAWB items.

SCI-QOL Items and parameters copyright ©2015 David Tulsky and Kessler Foundation. All Rights Reserved. Neuro-QOL items copyright ©2015 David Cella. All Items and Scales should be accessed and used through the corresponding author or http://www.assessmentcenter.net. Do not modify items without permission from the copyright holder.

### Dimensionality

Using CFA, a unidimensional model was observed (CFI = 0.947; RMSEA = 0.094). *R*^2^ values for 28 items were greater than 0.40 and none were less than 0.40. In terms of local dependence, no item pairs exhibited residual correlations >|0.20|). Eigenvalue ratio (first to second) was 15.1.

### Irt parameter estimation and model Fit

**Figure 1:**
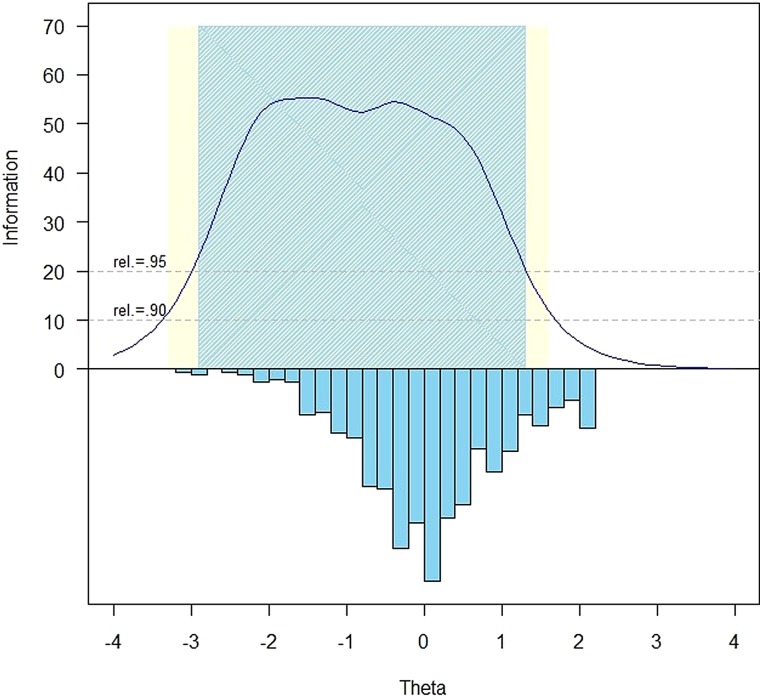
SCI-QOL positive affect & well-being item bank information and precision.

Slopes ranged from 1.81 to 3.66, with thresholds ranging from –3.15 to 1.79.The measurement precision in the theta range between –2.9 and 1.2 is roughly equivalent to a classical reliability of 0.95 or better (Fig. 1).

The S-X^2^ model fit statistics were examined using the IRTFIT macro program. All items had adequate or better model fit statistics (P > 0.05), with marginal reliability^52^ equal to 0.969 and no item pairs were flagged (residual correlation >|0.2|) for local dependence.

### Differential item functioning (DIF)

DIF was examined using *lordif*^53^ for six categories: age (≤49 vs. ≥50), sex (male *n* = 559 vs. female *n* = 158), education (some college and lower *n* = 523 vs. college degree and above *n* = 194), diagnosis (tetraplegia *n* = 388 vs. paraplegia *n* = 325), injury severity (incomplete *n* = 374 vs. complete *n* = 339), and time post injury (<1 year *n* = 196 vs. >1 year *n* = 521). Items were flagged for possible DIF when the probability associated with the χ^2^ test was <0.01 and the effect size measures (McFadden's pseudo *R*^2^) >0.02, which is a small but non-negligible effect. Overall, 12 items were flagged for DIF in at least one category based on the chi-square test; however, when the effect size measures were examined, the DIF was negligible and all 28 items were retained in the final, calibrated item bank.

### Transformation to Neuro-QOL metric

Given the availability of 22 verbatim Neuro-QOL items to use as ‘anchors’, the SCI-QOL PAWB item bank IRT parameters were transformed to the Neuro-QOL metric. In this way, the SCI-QOL parameters which yield scores based on an SCI population (e.g. the mean of 50 represents the mean of a large sample of individuals with SCI) were transformed to the Neuro-QOL metric (i.e. so that the mean of 50 will represent the mean of the general population) to ensure that SCI-QOL and Neuro-QOL PAWB scores are directly comparable. As reported above, before transformation, slopes ranged from 1.81 to 3.66, with thresholds ranging from −3.15 to 1.79. The sample mean was 51.15 and the standard deviation (SD) was 9.61. After transformation, slopes range from 2.25 to 4.54 and thresholds range from −2.18 to 1.79 (see Table 3). When scored using the transformed parameters, the sample mean was 54.47 and the SD was 7.92.

**Table 3:** Positive affect and well-being items and item bank parameters

Item ID	Item Stem	Item Response Theory Calibration Statistics				
		Slope	Threshold 1	Threshold 2	Threshold 3	Threshold 4
NQPPF01	I felt happy about the future	3.95301	−1.14949	−0.53845	0.22284	0.87856
NQPPF02	I was able to enjoy life	3.54867	−1.49391	−0.77118	−0.01380	0.82889
NQPPF03	I felt a sense of purpose in my life	4.04474	−1.30455	−0.66602	0.00531	0.62264
NQPPF04	I could laugh and see the humor in situations	2.34125	−2.18607	−1.34873	−0.38393	0.65077
NQPPF05	I was able to be at ease and feel relaxed	2.62732	−1.56956	−0.70503	0.26616	1.14028
NQPPF06	I looked forward with enjoyment to upcoming events	3.20216	−1.68157	−0.79648	−0.03707	0.71524
NQPPF07	Many areas of my life were interesting to me	3.16476	−1.57612	−0.68029	0.18464	0.90224
NQPPF08	I felt emotionally stable	2.74422	−1.66864	−1.03842	−0.18287	0.76859
NQPPF11	I felt confident	3.06251	−1.52046	−0.75344	0.10620	0.95162
**NQPPF12**	**I felt hopeful**	**3.79944**	−**1.35445**	−**0.96665**	−**0.09410**	**0.70839**
NQPPF13	I had a good life	3.61491	−1.39578	−0.79674	−0.04910	0.69237
**NQPPF14**	**I had a sense of well-being**	**3.61408**	−**1.43705**	−**0.75512**	**0.06521**	**0.87276**
NQPPF15	My life was satisfying	3.67461	−1.31124	−0.61968	0.20983	1.02506
**NQPPF16**	**I had a sense of balance in my life**	**3.44348**	−**1.30183**	−**0.58562**	**0.32749**	**1.05953**
**NQPPF17**	**My life had meaning**	**4.01958**	−**1.23286**	−**0.69150**	**0.01161**	**0.57683**
NQPPF18	My life was peaceful	2.26219	−1.57107	−0.75030	0.27463	1.24068
**NQPPF19**	**My life was worth living**	**3.68923**	−**1.58122**	−**1.24680**	−**0.57849**	**0.08843**
**NQPPF20**	**My life had purpose**	**3.88416**	−**1.28004**	−**0.75537**	−**0.07167**	**0.47673**
**NQPPF21**	**I was living life to the fullest**	**3.18129**	−**0.94064**	−**0.26560**	**0.45139**	**1.04140**
**NQPPF22**	**I felt cheerful**	**3.33932**	−**1.65641**	−**0.85367**	**0.16288**	**1.11203**
NQPPF23	In most ways my life was close to my ideal	2.26028	−0.65162	0.02829	0.93254	1.78892
NQPPF24	I had good control of my thoughts	2.25262	−1.95111	−1.32336	−0.28170	0.81310
NQPPF26	Even when things were going badly, I still had hope	2.83023	−1.80193	−1.20679	−0.29079	0.55586
PPF_29	I was optimistic about the future	2.62517	−1.46749	−0.76172	0.11161	0.89749
**PPF_30**	**I thought positively about my future**	**4.54921**	−**1.16245**	−**0.64926**	**0.06604**	**0.69873**
**PPF_32**	**I was thankful to be alive**	**3.72859**	−**1.55655**	−**1.14642**	−**0.47812**	**0.05993**
PPF_33	I was proud of everything that I have overcome	2.68008	−1.76538	−1.06275	−0.22474	0.52363
PPF_34	I had a positive attitude about living with my injury	2.97887	−1.36136	−0.83589	−0.02627	0.85965

*Context for all items was: ‘Lately’. Response set was: 1 = Never/2 = Rarely/3 = Sometimes/4 = Often/5 = Always.

**Bold Font** indicates the items selected for the short form 10a.

With the exception of item NQPPF01, all items beginning with ‘NQPPF’ are final Neuro-QOL PAWB items.

SCI-QOL Items and parameters copyright © 2015 David Tulsky and Kessler Foundation. All Rights Reserved. Neuro-QOL items copyright © 2015 David Cella. All Items and Scales should be accessed and used through the corresponding author or http://www.assessmentcenter.net. Do not modify items without permission from the copyright holder.

### Short form selection and mode of administration

Once the SCI-QOL Positive Affect & Well-being item bank was finalized, all items and parameters were programmed into the Assessment Center^SM^^54^ platform and the bank is now freely available as a CAT. Since the purpose of calibrating items using IRT is that only a subset of items needs to be administered from a given bank in order to estimate an individual's score, there is flexibility as to how the items are selected and administered. On the Assessment Center platform, the CAT administration parameters can be modified to reduce standard error variance (e.g. maximize reliability), or to reduce test burden. There is also a predetermined static short form that can be downloaded. Finally, the individual items are present and could be selected if the end user wanted to administer a specific item. These administration options are reviewed below.

The SCI-QOL utilizes the same default CAT discontinue criteria as Patient Reported Outcomes Measurement Information System (PROMIS); namely, the CAT minimum number of items to administer is four and the maximum is 12 with a maximum standard error of 0.3. In other words, in the default settings, the CAT will always administer at least 4 items, then will discontinue when the standard error of the individual's score estimate drops below 0.3 or a maximum of 12 items is reached (and the standard error variance criterion cannot be met).

Alternatively, the user could change the ‘discontinue criteria’ of the CAT so that it will administer additional items and obtain a more precise assessment of functioning. For instance, if the user selected an option that the CAT administers a minimum of 8 items before discontinuing, a lengthier test would be administered, but a more reliable score will be obtained. In some cases, greater precision over test burden is desirable based on factors such as resource allocation where specificity is critical.

However, in some cases it is neither possible (e.g. internet unavailable) nor practical (e.g. laptop/tablet computer equipment beyond budget of project) to administer items via CAT. To address this need, the positive affect and well-being and other SCI-QOL item banks are also available as short forms The project investigators utilized psychometric and clinical input to develop a fixed, 10-item short form version of the positive affect and well-being item bank. The goal of the short form selection process was to include the most informative items across a wide range of ‘difficulty’, or amount of the underlying trait. Since all items are calibrated on the same metric, scores on the short form are directly comparable to those on the CAT or full item bank. The correlation of the short form and various CATs with the full bank are given in Table 3. Short forms may be administered directly within Assessment Center, or may be downloaded for administration by paper and pencil, or an alternate data capture platform or system. Individual investigators or clinicians could also develop additional, custom short forms, which could then be scored on the same IRT-based metric with the help of a psychometrician.

**Figure 2:**
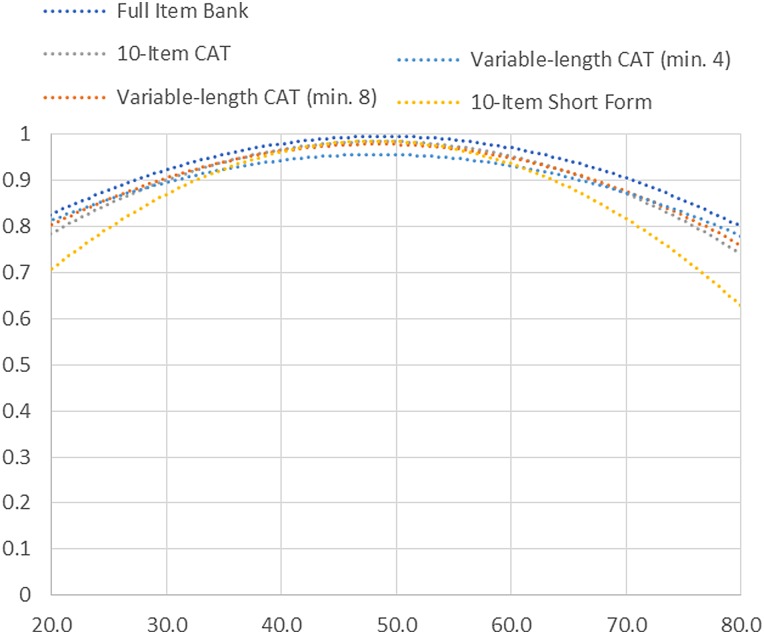
SCI-QOL PAWB: Measurement Reliability by T-score and assessment method.

To determine the degree of measurement precision and error for these assessments, we compared the reliability of the full bank, 8-item short form, variable-length CAT with the default minimum of 4 items, and variable-length CAT with a minimum of 8 items Table 4 presents the mean number of items presented and standard deviation (CATs only), T-score range, and standard error range for each of the various administration modes; Table 5 gives the breadth of coverage for all modes of administration. Additionally, reliability curves for the full bank, short form, variable length CAT (minimum of 4 items) and fixed-length CAT (8 items) are displayed in Fig. 2.

**Table 4:** Accuracy of variable- and fixed-length CAT and 10-item short form: correlations with full-bank score

Mode	*N*	# Items Admin			Max	%Min	%Max	Correlation with full bank
		Mean	SD	Min				
Variable-length CAT (min 4)	717	5.10	2.38	4	12	73.32%	8.80%	0.950
Variable-length CAT (min 8)	717	8.39	1.17	8	12	89.53%	9.22%	0.975
10-item fixed-length CAT	717	N/A	N/A	10	10	N/A	N/A	0.981
10-item short form	717	N/A	N/A	10	10	N/A	N/A	0.976

**Table 5:** Breadth of coverage for variable length CAT, fixed length CAT, 10-item short form, and full item bank

Mode	N	T Score				Standard Error	
		Mean±SD	Range	% Ceiling	% Floor	Mean±SD	Range
Variable-length CAT (min 4)	716	54.35 ± 7.94	26.66–72.23	3.49%	0.14%	0.251 ± 0.0517	0.202–0.462
Variable-length CAT (min 8)	716	54.43 ± 7.92	26.66–72.23	3.49%	0.14%	0.203 ± 0.0672	0.148–0.462
10-item fixed-length CAT	716	54.46 ± 7.95	23.27–71.86	4.47%	0.14%	0.191 ± 0.0759	0.134–0.467
10-item short form	716	54.41 ± 7.70	26.7–68.6	9.92%	0.14%	0.213 ± 0.0946	0.140–0.480
Full bank	716	54.49 ± 7.92	28.2–73.2	3.07%	0.14%	0.139 ± 0.0714	0.100–0.450

#### Scoring

SCI-QOL Positive Affect and Well-being scores are standardized on a T-metric, with a mean of 50 and a standard deviation of 10; this is based on the SCI-QOL calibration data; that is, a mean of 50 reflects the mean of an SCI population rather than the general population. All CAT administrations of the SCI-QOL Positive Affect and Well-being item bank are automatically scored by Assessment Center. When administering the short form, whether via Assessment Center, paper and pencil, or another data capture platform, an individual must complete all 10 component items in order to receive a score. The raw score for the short form is computed by simply summing the response scores for the individual component items and identifying the T-score and associated standard error for each raw score value is given in Table 6.

**Table 6:** Raw score to T-score conversion table for PAWB SF10a

Raw score	T-score	Standard error
10	26.7	4.1
11	30.5	2.7
12	32.2	2.4
13	33.5	2.1
14	34.6	1.9
15	35.5	1.8
16	36.3	1.8
17	37.1	1.7
18	37.8	1.7
19	38.5	1.7
20	39.2	1.6
21	39.9	1.6
22	40.6	1.6
23	41.2	1.6
24	41.9	1.6
25	42.5	1.6
26	43.2	1.7
27	43.9	1.7
28	44.6	1.7
29	45.3	1.7
30	46.0	1.7
31	46.7	1.7
32	47.4	1.7
33	48.1	1.7
34	48.9	1.7
35	49.6	1.7
36	50.3	1.7
37	51.1	1.7
38	51.8	1.7
39	52.6	1.7
40	53.3	1.7
41	54.1	1.7
42	55.0	1.8
43	55.8	1.8
44	56.8	1.9
45	57.8	2.0
46	58.9	2.1
47	60.2	2.2
48	61.8	2.5
49	63.9	3.0
50	68.6	4.7

#### Reliability

As a part of the reliability study described in the Tulsky *et al.*^50^ methods paper in this issue, we compared PAWB scores at Baseline with those from the 1-2 week retest assessment. In a sample of 245 individuals with SCI, Pearson's *r* = 0.78 and ICC (2,1) = 0.78 (95% CI = 0.72 to 0.82).

## Discussion

As reflected in the Introduction, an emerging literature on quality of life suggests that emotional experiences related to positivity, well-being, growth, self-efficacy, self-esteem, spirituality, optimism, and hope exist post-SCI, but the field is restricted by the limited measurement tools currently available to assess these constructs with this population.^9–11^ We therefore developed the SCI-QOL Positive Affect and Well-being item bank to assess the positive dimensions of emotional functioning after SCI and to increase valid and reliable measurement and enhance operationalization of these constructs. Our approach incorporated the benefits of using an already well-validated measure for individuals with comparable neurological problems (Neuro-QOL), and customizing it specifically for use with individuals with SCI. It is notable that five of the 28 items that comprise the final bank were newly generated; the other 23 were originally drawn from Neuro-QOL. This may suggest that among persons with neurological disorders there is a shared experience of positive affect and well-being that the items reflect. This item bank complements the SCI-QOL Resilience and Self-Esteem item banks by capturing emotional states that may be more situationally-dependent and naturally fluctuate. This distinction is relevant as each construct may contribute differentially to psychological outcomes post-injury. Moreover, this is the first known initiative of its kind to create a robust measure of positive affect and well-being after SCI that can help to distinguish positive affect and well-being from other similar constructs such as resilience. As such, this new item bank can also help to further develop conceptual models of adjustment after SCI, including the trajectory of positive affect and well-being over time.

The inclusion of 22 verbatim Neuro-QOL items and transformation to the Neuro-QOL metric has yielded a tool that is optimized for SCI in terms of item inclusion and order of administration, while simultaneously yielding scores that are directly comparable to Neuro-QOL Positive Affect and Well-being scores and therefore the general population. This linkage greatly increases the opportunities for cross-condition and cross-study comparison of treatments, interventions, or outcomes.

The use of IRT to calibrate the SCI-QOL Positive Affect and Well-being items has yielded several administration options, including short forms and CAT. If a user's goal is to optimize reliability, especially at the ceiling and floor of the distribution, we would recommend administering the Positive Affect and Well-being item bank as a CAT. In cases where it may not be feasible or practical to administer items via CAT/Assessment Center, or if having participants answer the same subset of items is necessary to answer a given research question, we would recommend short form administration. An additional administration option is to administer both the CAT and any short form items not included in the CAT by using the ‘no duplicates’ option in Assessment Center. The flexibility of methods to administer the SCI-QOL Positive Affect and Well-being item bank also provides scientists and clinicians with an efficient and accessible way to integrate the measurement of positive affect and emotional well-being that is specifically relevant to SCI into research and, ultimately, clinical practice. Future directions include evaluation of positive affect and well-being as a moderator of a variety of outcomes following SCI, most notably emotional outcomes such as depression and anxiety.

### 

#### Study limitations

We acknowledge that the RMSEA value for the final item bank is 0.094 which is greater than the 0.08 ideal. However, values below 0.10 are typically considered acceptable for CAT applications, and in this case the tradeoff of eliminating additional items to slightly improve fit to a unidimensional model was not deemed worthwhile. Further, a potential limitation of the study is that due to the linkage to the Neuro-QOL metric, the SD of the sample has been reduced (i.e. from an SD of ∼10 to ∼8). The decreased standard deviation may be a result of linking to the general population or may simply be due to the nature of the measures.

## Conclusion

The final SCI-QOL Positive Affect and Well-being item bank contains 28 IRT-calibrated items Due to the flexibility of IRT-based measures, the use of CATs is also possible with this item bank, which enables researchers and clinicians to administer only the most precise and informative items based on an individual's responses. This has implications for the use of such innovative applications in emotional responses to injury in post-acute care settings. Our formative development work using focus groups and interviews supports previous quality of life literature suggesting the existence of characteristics related to positive affect and wellbeing post-SCI, and has expanded our knowledge of these constructs and their utility and importance with this population. Greater consistency of measurement across samples and settings will also strengthen our understanding of positive affect and well-being after injury and inform conceptual models.

## Disclaimer statements

**Contributors** All authors have contributed significantly to the design, analysis and writing of this manuscript. The contents represent original work and have not been published elsewhere. No commercial party having a direct financial interest in the results of the research supporting this article has or will confer a benefit upon the authors or upon any organization with which the authors are associated.

**Funding** This study was supported by National Institutes of Health grant number 5R01HD054659 (Eunice Kennedy Shriver National Institute of Child's Health and Human Development/National Center on Medical Rehabilitation Research and the National Institute on Neurological Disorders and Stroke).

**Conflicts of interest** No commercial party having a direct financial interest in the results of the research supporting this article has or will confer a benefit upon the authors or upon any organization with which the authors are associated.

All SCI-QOL items and parameters are © 2015 David Tulsky and Kessler Foundation. All rights reserved. Neuro-QOL items © David Cella. All items are freely available to the public via the Assessment Center platform (www.assessmentcenter.net). There are currently no plans for Dr. Tulsky or Kessler Foundation to benefit financially from the use of the copyrighted material.

**Ethics approval** The Institutional Review Board at each site reviewed and approved this project.
